# Comparative analyses of eight complete plastid genomes of two hemiparasitic *Cassytha* vines in the family Lauraceae

**DOI:** 10.3389/fgene.2023.1192170

**Published:** 2023-12-13

**Authors:** Qun-Fei Yu, Yun-Hong Tan, Wen-Bin Yu, Shi-Ting Yang, Jie-Peng Huang, Marcos A. Caraballo-Ortiz, Chao Liu, Yu Song

**Affiliations:** ^1^ Center for Integrative Conservation and Key Laboratory of Tropical Plant Resources and Sustainable Use, Xishuangbanna Tropical Botanical Garden, Chinese Academy of Sciences, Mengla, Yunnan, China; ^2^ University of Chinese Academy of Sciences, Beijing, China; ^3^ Southeast Asia Biodiversity Research Institute, Chinese Academy of Sciences, Yezin, Nay Pyi Taw, Myanmar; ^4^ Key Laboratory of Ecology of Rare and Endangered Species and Environmental Protection (Ministry of Education) and Guangxi Key Laboratory of Landscape Resources Conservation and Sustainable Utilization in Lijiang River Basin, Guangxi Normal University, Guilin, Guangxi, China; ^5^Department of Botany, National Museum of Natural History, Smithsonian Institution, Washington, DC, United States; ^6^ College of Biological Resource and Food Engineering, Qujing Normal University, Qujing, China

**Keywords:** hemiparasitic genus, chloroplast, *Cassytha*, *Eusideroxylon*, Laurales

## Abstract

*Cassytha* is the sole genus of hemiparasitic vines (ca. 20 spp.) belonging to the Cassytheae tribe of the Lauraceae family. It is extensively distributed in tropical and subtropical regions. In this study, we determined the complete plastid genome sequences of *C. filiformis* and *C. larsenii*, which do not possess the typical quadripartite structure. The length of *C. filiformis* plastomes ranged from 114,215 to 114,618 bp, whereas that of *C. larsenii* plastomes ranged from 114,900 to 114,988 bp. Comparative genomic analysis revealed 1,013 mutation sites, four large intragenomic deletions, and five highly variable regions in the eight plastome sequences. Phylogenetic analyses based on 61 complete plastomes of Laurales species, 19 ITS sequences, and *trnK* barcodes from 91 individuals of *Cassytha* spp. confirmed a non-basal group comprising individuals of *C. filiformis*, *C. larsenii*, and *C. pubescens* in the family Lauraceae and proposed a sister relationship between *C. filiformis* and *C. larsenii*. Further morphological comparisons indicated that the presence or absence of hairs on the haustoria and the shape or size of fruits were useful traits for differentiating *C. filiformis* and *C. larsenii*.

## 1 Introduction

The genus *Cassytha* L., belonging to the family Lauraceae, encompasses more than 20 hemiparasitic vines found in tropical and subtropical regions worldwide (Weber, 1981). Among these regions, Africa, Asia, and Australia host three, four, and nineteen species, respectively, and Australia stands as the center of species diversity for *Cassytha*. The dodder laurel (*C. filiformis* L.), which is the only pantropical species of *Cassytha*, is used as a medicinal plant in various regions, such as Bahamas, China, Indonesia, Nigeria, and the West Indies. It contains alkaloids, flavonoids, phenol, saponin, terpenoids, and tannin (Burkill, 1995; Tsai et al., 2008; Brophy et al., 2009; Adamu et al., 2017; Nazar et al., 2019). *Cassytha*, as a member of plants that are adapted to grow in open environments (Jordan et al., 2014; Carpenter et al., 2015), possesses tiny triangular leaves and filiform stems that feature stomata located on any part of both cuticles (Awang et al., 2018). The cuticular characters, along with the glabrous or pubescent stem and petal, have been utilized to distinguish different *Cassytha* species (Kokubugata et al., 2012). Species identification has often been challenging due to the greatly reduced plant populations and similar habitats. For instance, in 1971, Hatusima described the stems of *C. filiformis* as thin and reddish. However, later *C. filiformis* was redefined as a Ryukyu endemic taxon, *C. pergracilis*, by Hatusima in 1976 (Hatusima, 1971; Hatusima, 1976). Over the last two decades, molecular diagnostic methods for *Cassytha* species have continuously improved.

In order to distinguish *Cassytha* species, molecular analyses have utilized a partial sequence of the *trnK* intron (*trnK*). Three separate analyses have been conducted, with differing results. Rohwer and Rudolph (2005) found *C. ciliolata* was sister to *C. pubescens*, although without bootstrap support. Meanwhile, Wang et al. (2010) identified *C. ciliolata* as sister to *C. filiformis*, with *C. pubescens* as the subsequent sister species, and *C. melantha* as the most basal species with high support. Kokubugata et al. (2012) conducted a more extensive sampling, which revealed a clade comprising *C. pubescens*, *C. muelleri*, and *C. rufa* as sister to another clade containing *C. filiformis*, *C ciliolata*, *C. capillaris*, and *C. pergracilis*. *C. glabella* was identified as the next sister group, followed by *C. melantha*.

At the genus level, previous molecular phylogenetic analyses based on diverse datasets have revealed incongruent placements of *Cassytha* species in the family Lauraceae. Rohwer’s original research Rohwer, (2000) employed the plastid marker *trnK* and sampling 48 species, which estimated that *C. ciliolata* formed a sub-basal clade within the family Lauraceae. A year later, Chanderbali et al. (2001) utilized plastid sequences, including *psbA-trnH*, *rpl16*, *trnL-trnF*, and *trnT-trnL*, as well as 26 S nuclear ribosomal DNA (nrDNA), to reconstruct phylogenetic relationships among 77 species in the family Lauraceae. Their analyses suggested that *C. filiformis* and *C. pubescens* were most closely related to *Neocinnamomum mekongense* (Hand.-Mazz.) Kosterm. Subsequently, a third study by Rohwer and Rudolph (2005), based on *trnK* sequences of 49 species, indicated that the monophyletic *Cassytha* group was not sub-basal within the family Lauraceae; Wang et al. (2010) used plastid sequences (*psbA-trnH* and *trnK*) and nrDNA to reconstruct the phylogenetic relationships of *Neocinnamomum* and showed a close relationship between the genera *Cassytha* and *Neocinnamomum*. However, Li et al. (2016) utilized nuclear gene *RPB2* fragment and ITS to reconstruct the phylogenetic relationships of *Caryodaphnopsis* and indicated an independent clade of *C. filiformis*.

Plastid genome sequencing has proven to be a valuable tool for elucidating the phylogenetic relationships of Angiosperm plants (Li et al., 2019; Li et al., 2021; Dong et al., 2022b). In order to determine the phylogenetic location of nineteen genera, Song et al. (2017b) conducted comparisons based on 47 Lauraceae plastid genomes and found support for the monophyletic clade of *Cassytha* within the family Lauraceae. Subsequently, a second study by Song et al. (2020) utilized a higher sampling of plastomes for 97 species and reconstructed a monophyletic Lauraceae clade that included the independent *Cassytha* subclade. This robust monophyletic *Cassytha* group was further supported by the analysis of complete nrDNA sequences with a length of 6,281 bp (Liu Z. F. et al., 2021).

In this study, we selected eight individuals of two *Cassytha* species to obtain their complete plastid genomes and nrDNA sequences. By comparing these sequences, we aim to answer three questions. Firstly, which types of mutation events occurred in the plastid genomes of *Cassytha*? Secondly, is there any highly variable region in the plastid genomes of *Cassytha* for DNA barcoding? Finally, what is the phylogenetic placement of *C. larsenii*? Comparisons were made with the taxonomic character data between *C. filiformis* and *C. larsenii*.

## 2 Materials and methods

### 2.1 Plant materials

**FIGURE 1:**
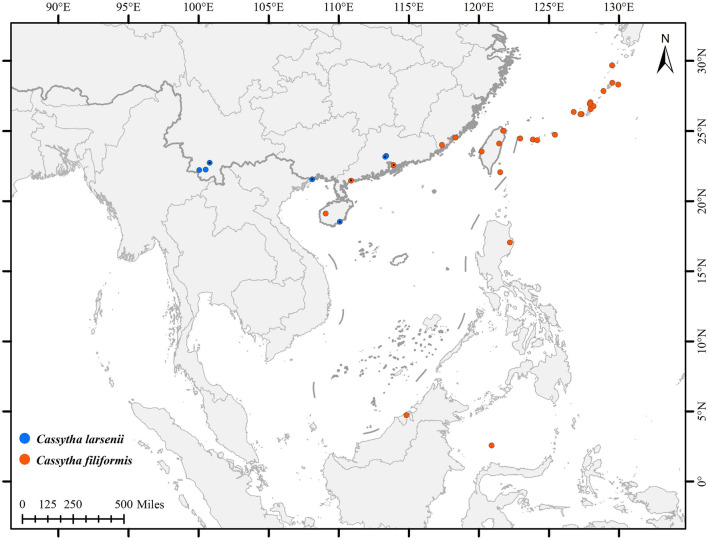
Distribution of *C. filiformis* and *C. larsenii*. Blue pots indicate *C. larsenii* and red pots indicate *C. filiformis*.

In this study, a total of eight individuals from two species, *C. filiformis* and *C. larsenii*, were sampled. Fresh stems of wild vines were collected from China and Puerto Rico, and quickly dried with silica gel (Table 1). Voucher specimens were deposited in the herbarium of Guangxi Normal University. The specimens were identified by Yun-Hong Tan (Xishuangbanna Tropical Botanical Garden, CAS) and Yu Song (Guangxi Normal University). Furthermore, plastid genome sequences of related taxa of Lauraceae were downloaded from Lauraceae Chloroplast Genome Database (LCGDB) (https://lcgdb.wordpress.com) and GenBank of NCBI (https://www.ncbi.nlm.nih.gov) and a total of 61 taxa from 27 genera of Laurales were included. In addition, the partial *trnK* intron, including *matK* gene sequences, of eight newly sequenced and 83 individuals of *Cassytha* obtained from the NCBI database were used to conduct phylogenetic analyses (Figure 1; Supplementary Figure S1).

**TABLE 1:** Vouchers and accession no. of individuals of the *Cassytha* sequenced in this study.

No	Species	Collection	Locality	Herbarium	Accession No.	Year
1	*Cassytha filiformis* L.	Chen Hui SY36646	Maoming, Guangdong, China	HITBC-BRG	OR766688	Oct 2020
2	*Cassytha filiformis* L.	Zhang Ting J1572	Nanwa, Shenzhan, China	KIB	OR766689	Jun 2019
3	*Cassytha filiformis* L.	Caraballo-Ortiz 3075	Little Cayman, Cayman Islands	PAC	OR766690	Apr 2012
4	*Cassytha filiformis* L.	Caraballo-Ortiz 3204	Guánica, Puerto Rico	PAC	OR766691	Oct 2012
5	*Cassytha larsenii* Kosterm.	Song Yu SY34990	Puer, Yunnan, China	HITBC-BRG	OR766692	Jul 2018
6	*Cassytha larsenii* Kosterm.	Song Yu SY37174	Guangzhou, Guangdong, China	HITBC-BRG	OR766693	Jul 2018
7	*Cassytha larsenii* Kosterm.	Zhang Ting F940	Lingshui, Hainan, China	KIB	OR766694	Jun 2019
8	*Cassytha larsenii* Kosterm.	Zuo Yunjuan Z2138	Dongxing, Guangxi, China	HITBC-BRG	OR766695	Nov 2020

### 2.2 Plastid genome sequencing and assembly

Whole-genomic DNA was extracted from the stem tissues using the CTAB method (Doyle and Doyle, 1987). Libraries were constructed with fragments of approximately 300 bp according to the manufacturer’s protocol (Illumina, San Diego, CA, United States). All samples were sequenced using the Illumina HiSeq 2,500 at Kunming Institute of Botany, Chinese Academy of Sciences (KIB, CAS). The plastid genomes were *de novo* assembled using the GetOrganelle pipeline under default settings (Jin et al., 2020; Dong et al., 2022a). The assembly quality of all plastid genomes was checked with Bandage software (Wick et al., 2015).

### 2.3 Genome annotation

The newly assembled plastid genomes were annotated using the GeSeq application, followed by manual verification using the Geneious software (Kearse et al., 2012; Tillich et al., 2017). The annotation sequences and their corresponding information were then submitted to Genbank and assigned accession numbers OR766688 to OR766695. Finally, the physical map of the annotated plastid genomes was drawn using the online Chloroplot program (https://irscope.shinyapps.io/Chloroplot/).

### 2.4 Plastome sequence divergence and microstructural mutation analysis

In order to compare the divergence within eight newly assembled plastid genomes, the online mVISTA program in Shuffle-LAGAN mode (https://genome.lbl.gov/vista/mvista/submit.shtml) was employed. Additionally, the plastid genome sequence’s nucleotide diversity (Pi) was estimated using DnaSP, with a step size of 200 bp and a window length of 600 bp for sliding window analysis (Rozas et al., 2017). The number and position of Indel and single-nucleotide polymorphism (SNP) events were determined by manual statistics and analyzed in the aligned eight plastid genome sequences of two *Cassytha* species.

### 2.5 Phylogenetic analyses

To determine the phylogenetic relationships within *Cassytha* and its relatives, the eight newly assembled plastid genomes were compared to the other 53 Laurales species, with *Illigera celebica* (LAU00199) and *I. grandiflora* (LAU00198) selected as outgroups. The sequences were aligned using MAFFT (Katoh et al., 2019) and manually adjusted in BioEdit (Hall et al., 2011). Maximum likelihood (ML) analysis was conducted using IQ-tree v2, and the best-fit model was determined using ModelFinder (Kalyaanamoorthy et al., 2017), with the GTR + F + I + G4 model and a bootstrap value of 1,000 (Minh et al., 2020). Then, the *trnK* and ITS sequences of the eight sequenced *Cassytha* individuals were intercepted and aligned with available *trnK* and ITS sequences from GenBank. A previous study showed *C. melantha* was basal to other *Cassytha* taxa based on extensive sampling (Kokubugata et al., 2012). Consequently, *C. melantha* was used as an outgroup in phylogenetic analyses. The best-fit DNA substitution models were chosen as TPM3uf + I + G (*trnK* data matrix) and TIM3+I (ITS data matrix) in jmodeltest v.2.1 (Darriba et al., 2012). Bayesian inference (BI) was performed for ten million generations, sampling every 1,000 generations in MrBayes v.3.2, with independent Markov chain Monte Carlo (MCMC) chains (Ronquist et al., 2012). The first 25% of the trees were discarded as burn-in, and the remaining trees were used to generate a majority-rule consensus tree. The MCMC output was examined and the effective sample size (ESS) values were above 200. Finally, the generated trees were visualized and adjusted using FigTree software (https://tree.bio.ed.ac.uk).

### 2.6 Morphological analyses

The pan-tropical *C. filiformis* species are the most widely studied compared with other *Cassytha* species, but the morphology of *C. larsenii* has not been fully reported to date. To this end, the morphological characters of two *Cassytha* species were observed by stereoscopic microscope (SM) and scanning electron microscope (SEM). The following morphological characters were focused on: haustoria, stems, petals, and fruits. For stereoscopic microscope observation, the haustoria, stems, and flowers were placed upright on a flat, wet tissue paper and observed with Leica S8 APO, LAS v 4.8 collecting photographs. SEM observations of the materials proceeded in ZEISS EVO LS10 scanning electron microscope at the Public Technology Service Center, Xishuangbanna Tropical Botanical Garden, Chinese Academy of Sciences.

## 3 Results

### 3.1 Genome features

**FIGURE 2:**
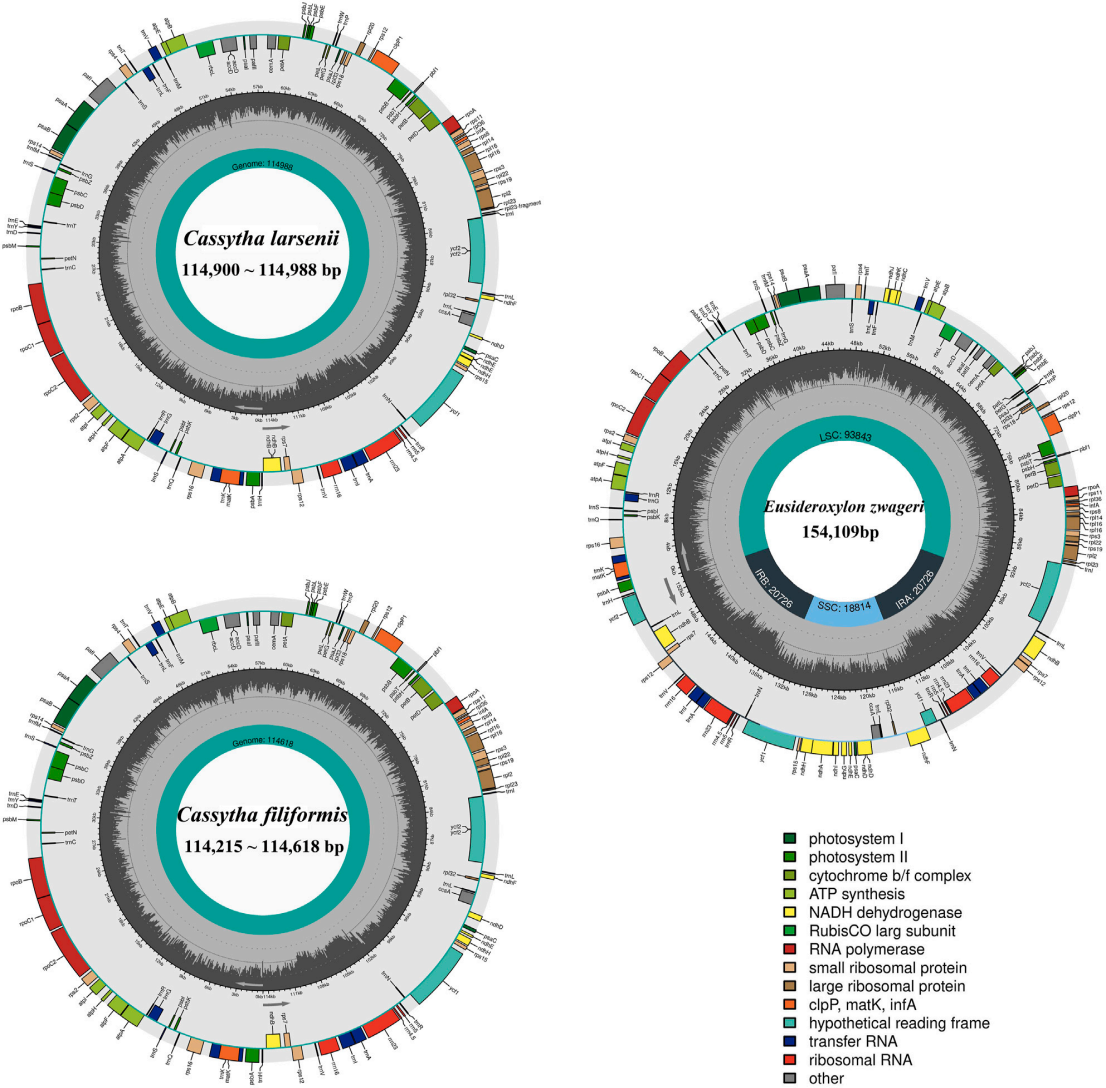
Plastome genome maps of two *Cassytha* species and *Eusideroxylon zwageri*. Genes displayed outside of the circle are transcribed counterclockwise, whereas those inside are transcribed clockwise. Diferent colors represent different functional gene groups. *Eusideroxylon zwageri* as a reference to IR/SSC/LSC.

All eight newly sequenced *Cassytha* plastomes were assembled into single circular molecules lacking the typical inverted repeat (IR) region and quadripartite structure (Figure 2). The size of the plastomes varied from 114,215 bp in *C. filiformis* growing in Puerto Rico (P304) to 114,618 bp in *C. filiformis* growing in Guangdong (SY6130), while the sizes range was from 114,900 bp in *C. larsenii* growing in Yunnan (SY9917) to 114,988 bp in *C. larsenii* growing in Guangxi (SY6156). The GC content was similar in all eight plastomes, with a value of 37.0%. Each plastome contained a total of 107 functional genes, including 73 protein-coding genes, 30 tRNA genes, and four rRNA genes (Table 2). Notably, unlike the NADH dehydrogenase (*ndh*) genes found in other sequenced Lauraceae plastomes, five *ndh* genes including ѱ*ndhB*, ѱ*ndhD*, ѱ*ndhE*, ѱ*ndhF*, and ѱ*ndhH* are pseudogenes, and six *ndh* genes, including *ndhA*, *ndhC*, *ndhG*, *ndhI*, *ndhJ*, and *ndhK* are absent in all eight *Cassytha* plastomes. In addition, we identified three genes *pafI*, *pafII*, and *pbf1* in all of these genomes.

**TABLE 2:** Summary of the complete plastomes of *Cassytha*.

	*C. filiformis*	*C. larsenii*
Individual number	4	4
Plastome size (bp)	114,215 ∼ 114,618	114,900 ∼ 114,988
GC content (%)	37.0	37.0
Number of genes	107	107
Protein encoding	73	73
tRNA	30	30
rRNA	4	4

### 3.2 Microstructural mutations

We assessed synteny and rearrangements in the eight *Cassytha* plastomes and found no large-scale recombination in the gene organization after verification. However, we manually detected 24 micro-inversions, ranging from 2 to 46 bp, in the regions of the *accD*, *accD-psaI*, *accD-rbcL*, *atpA-trnR*, *atpE-trnM*, *ccsA-psaC*, *clpP* intron, *psbA-trnH*, *petA-psbJ* (three regions), *petD-rpoA*, *petL-psbE*, *psbC-trnS*, *psbM-trnD*, *psbN-psbT*, *rpl32-trnL* (two regions), *rpoB-trnC*, *rps7-trnH*, *rps16* intron, *trnG-trnR*, and *ycf2* (two regions) (Table 3). Palindrome sequences in pairs with lengths of 3–23 bp were identified in the flanks of these inversions. Furthermore, we detected a total of 249 indels in the *Cassytha* species, which were classified into 195 simple sequences repeat (SSR) indels and 54 non-SSR indels (Supplementary Table S1).

**TABLE 3:** The predicted hairpin loops and stems of inversions in the eight plastomes of *Cassytha*.

No	Location	Loop motif	Size	Upstream stem sequence	Downstream stem sequence
1	*psbA-trnH*	tgat	4	tcaataccaaacttct	agaagtttggtattga
2	*rps16* intron	cttacttcctgaag	14	ttttttttttttttt	aaaaaaaaaaaaaaa
3	*trnG-trnR*	cac​act​ttc​cca​ttt​ccg​aaa​gga​aat​gga​atc​aga​ttg​tat​gtg	45	atttttttttttt	aaaaaaaaaaaat
4	*atpA-trnR*	aa	2	attttt	aaaaat
5	*rpoB-trnC*	caa	3	cat​gtt​ttt​ttt​ttt​ttt​tct​tt	aaa​gaa​aaa​aaa​aaa​aaa​aca​tg
6	*psbM-trnD*	ga	2	aaaaa	aaaaa
7	*psbC-trnS*	tcccacc	7	ggctcggcta	tagccgagcc
8	*atpE-trnM*	ttt​gtt​tat​aga​act​tat​ttg​ggt​att​gac​tcc	33	aacttattagatacc	ggtatctaataagtt
9	*accD-rbcL*	tag	3	tcttctatt	aatagaaga
10	*accD*	ttct	4	aactagaaaa	ttttctagtt
11	*accD-psaI*	tcc	3	ttccat	atggaa
12	*petA-psbJ*	ggaattttgcaccc	14	tttcgacacaagaaaa	ttttcttgtgtcgaaa
13	*petA-psbJ*	gga​gat​gat​ttc​ttg​aac​aaa​tag​aac​ttc​ttc​aat​gaa​cc	41	aaaaaaaaaaaaa	ttttttttttttt
14	*petA-psbJ*	ttt	3	gatg	catc
15	*petL-psbE*	atgccatggttactcc	16	aaatccaattctttt	aaaagaattggattt
16	*clpP* intron	ctt	3	ttttttttt	aaaaaaaaa
17	*psbN-psbT*	cgtatg	6	taa​ttg​aag​taa​tga​gcc​ccc	ggg​ggc​tca​tta​ctt​caa​tta
18	*petD-rpoA*	aaa	3	tcttttttttt	aaaaaaaaaga
19	*ycf2*	aa	2	tttcattc	gaatgaaa
20	*ycf2*	tc	2	caaatac	aattttg
21	*rpl32-trnL*	ttttttttttt	11	tctaactcttttttcttt	aaagaaaaaagagttaga
22	*rpl32-trnL*	ctt​tta​gat​ctt​tga​tac​caa​cca​aat​att​tat​aga​aac​ttt​ttg​g	46	tcattactacat	atgtagtaatga
23	*ccsA-psaC*	atc	3	aat	att
24	*rps7-trnH*	aac	3	agaatgaa	ttcattct

### 3.3 Plastome comparisons

**FIGURE 3:**
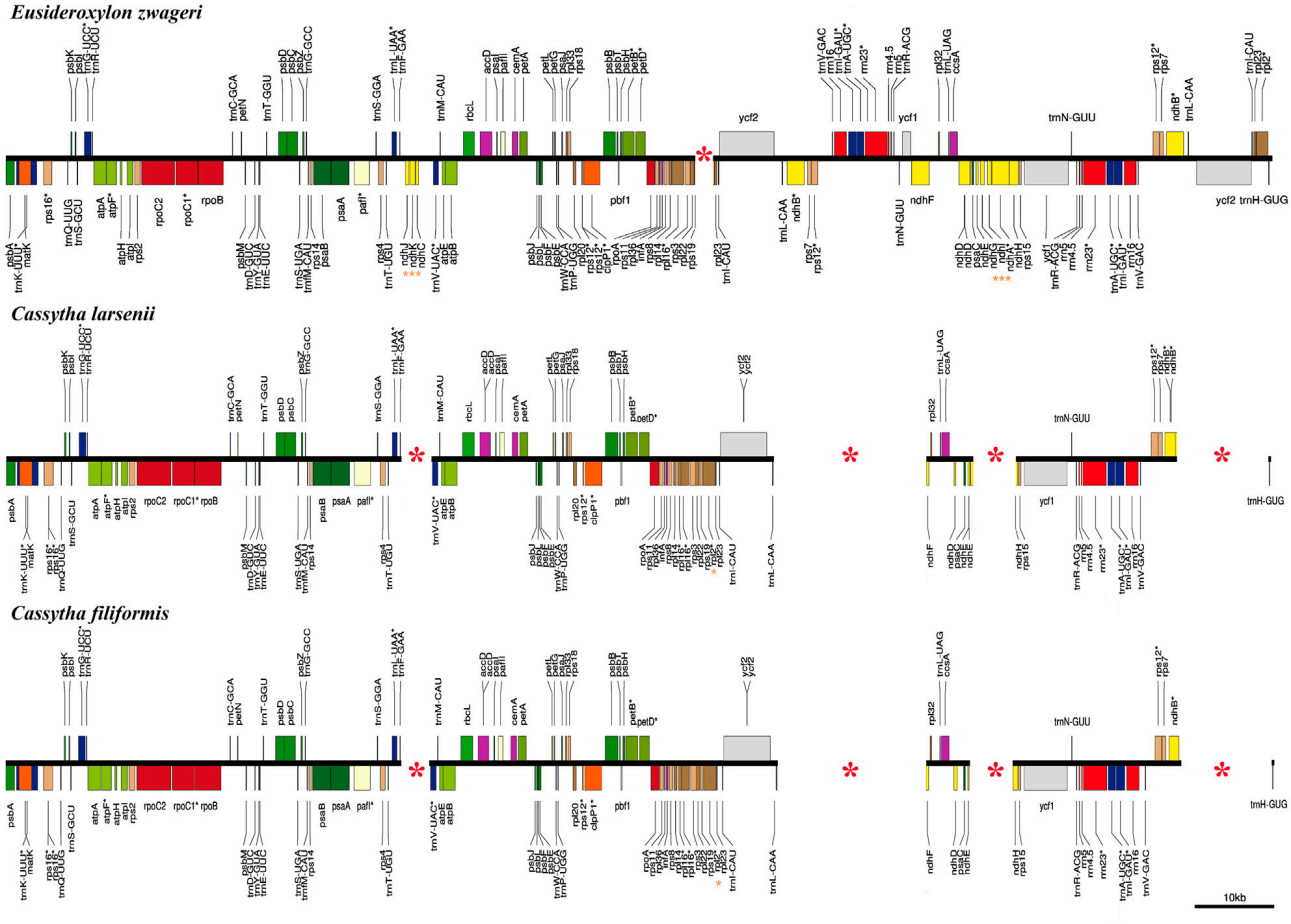
Comparison of the plastid genomes of *Eusideroxylon zwageri*, *C. filiformis* and *C. larsenii*. Missing segments and genes are marked with red asterisks.

In comparison to the previous published plastome of *Eusideroxylon zwageri* Teijsm. & Binn. (LAU00006), which is an early divergent species in the Lauraceae family, the *Cassytha* plastomes have four missing segments (Figure 3). These missing segments include a 4 kb fragment containing three *ndh* genes (*ndhC*, *ndhJ*, and *ndhK*), a 16 kb fragment flanked by *ndhB* and *ycf1*, a 4 kb fragment containing three *ndh* genes (*ndhA*, *ndhG*, and *ndhI*), and a 10 kb fragment flanked by *rpl2* and *trnL*-CAA. The missing segments with the length of 16 kb and 10 kb are located in the IRa and IRb regions of the *E. zwageri* plastome, respectively. The other two missing segments with a length of 4 kb are located in LSC and SSC regions of *E. zwageri* plastome, respectively. Additionally, when compared with the *Cassytha* plastome, a 1.5 kb segment containing the *rpl2* gene is absent from the IRa region of the *E. zwageri* plastome.

### 3.4 Divergence hotspot regions

**FIGURE 4:**
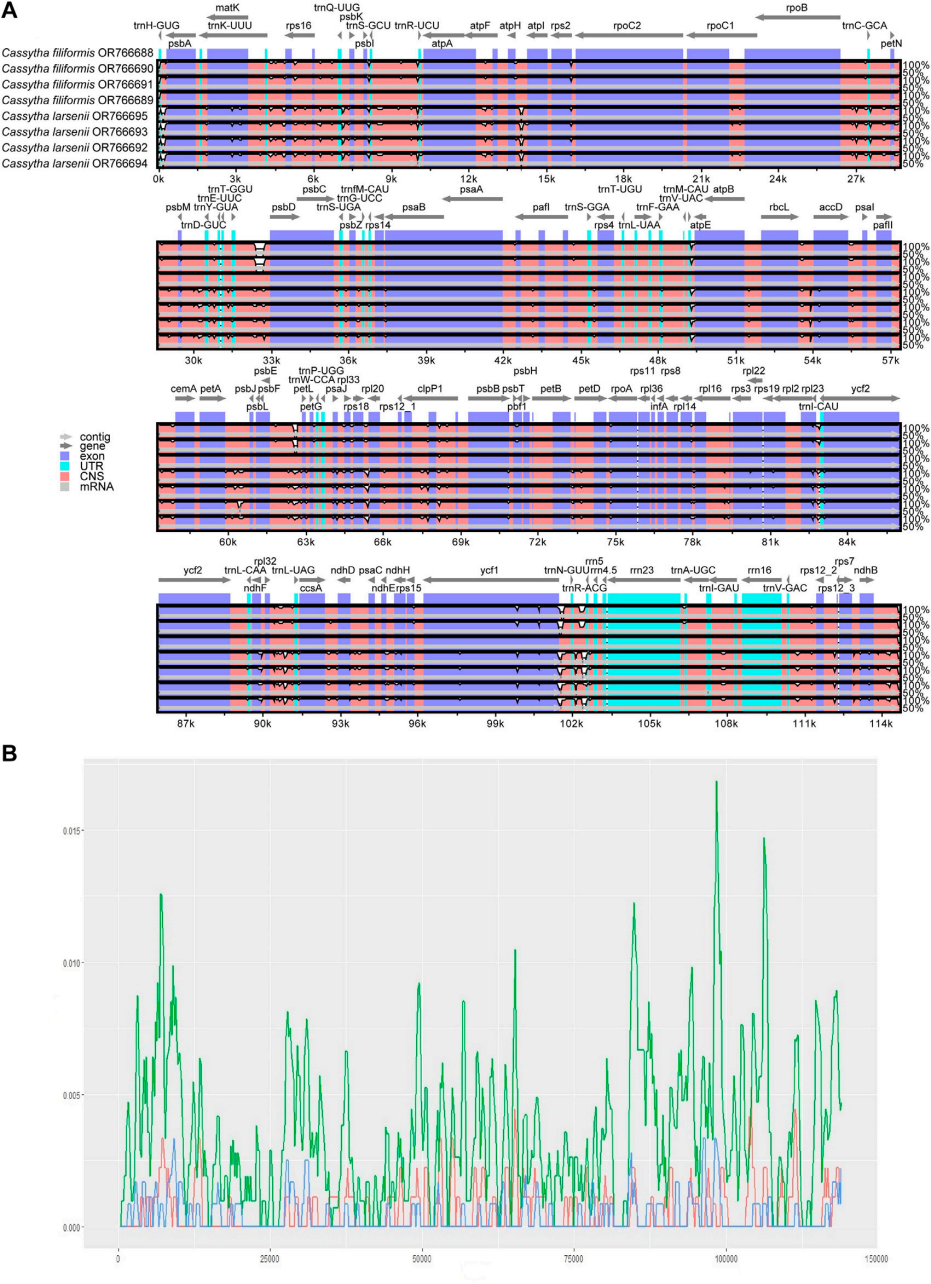
**(A)** Consistency alignment of the complete plastome of *C. filiformis* and *C. larsenii* using mVISTA.T-axis shows the percentage identity (50%–100%). Purple bars represent exon regions, blue bars represent untranslated regions (UTRs), pink bars represent noncoding sequences (CNS), gray bars represent mRNA. **(B)** Sliding-window analysis of the entire chloroplast genome of the two *Cassytha* species (green line), *C. filiformis* (blue line) and *C. larsenii* (red line). (window length: 600 bp, step size: 200 bp). X-axis: position of the window; Y-axis: nucleotide diversity of each window.

The mVISTA results show that the non-coding regions of the eight plastomes have higher levels of divergence than the coding regions. There are some gaps in the intergenic spacer regions of P304 and P306 of *C. filiformis* (Figure 4A). A total of 734, 200, and 193 SNP markers were detected in the plastomes of both *Cassytha* species, *C. larsenii* individuals, and *C. filiformis* individuals, respectively. The sequence divergence levels among the plastomes of *C. filiformis* and *C. larsenii* were determined (Figure 4B). Within the two *Cassytha* species, these values varied from 0 to 0.0168, with a mean of 0.0034. Within *C. filiformis*, these values varied from 0 to 0.0033, with a mean of 0.0004. Within *C. larsenii*, these values varied from 0 to 0.0053, with a mean of 0.0006. The pairwise nucleotide divergence values between two of the four plastomes varied from 0.000017 to 0.000967 in *C. filiformis* and from 0.000139 to 0.000715 in *C. larsenii*. The values between the two species varied from 0.005325 to 0.005839 (Table 4). These results indicate that the differences between the two species were more than six times higher than those among individuals. Five regions, namely, *trnQ*-*psbK*, *trnP*-*psaJ*, *rpl23*-*ycf2*, *ndhE*-*ndhH*, and *trnN*-*rrn5* were particularly highly variable between *C. filiformis* and *C. larsenii*.

**TABLE 4:** Pairwise nucleotide divergences of the eight plastomes of *Cassytha*.

No.	*Cassytha larsenii*				*Cassytha filiformis*			
	OR766695	OR766693	OR766694	OR766692	OR766690	OR766691	OR766688	OR766689
OR766695	—	0.000139	0.000304	0.000715	0.005778	0.005796	0.005831	0.005812
OR766693	0.000139	—	0.000218	0.000628	0.005691	0.005708	0.005769	0.005751
OR766694	0.000304	0.000218	—	0.000663	0.005761	0.005777	0.005839	0.005821
OR766692	0.000715	0.000628	0.000663	—	0.005325	0.005343	0.005435	0.005418
OR766690	0.005778	0.005691	0.005761	0.005325	—	0.000017	0.000951	0.000932
OR766691	0.005796	0.005708	0.005777	0.005343	0.000017	—	0.000967	0.000951
OR766688	0.005831	0.005769	0.005839	0.005435	0.000951	0.000967	—	0.000017
OR766689	0.005812	0.005751	0.005821	0.005418	0.000932	0.000951	0.000017	—

### 3.5 Phylogenetic reconstruction

**FIGURE 5:**
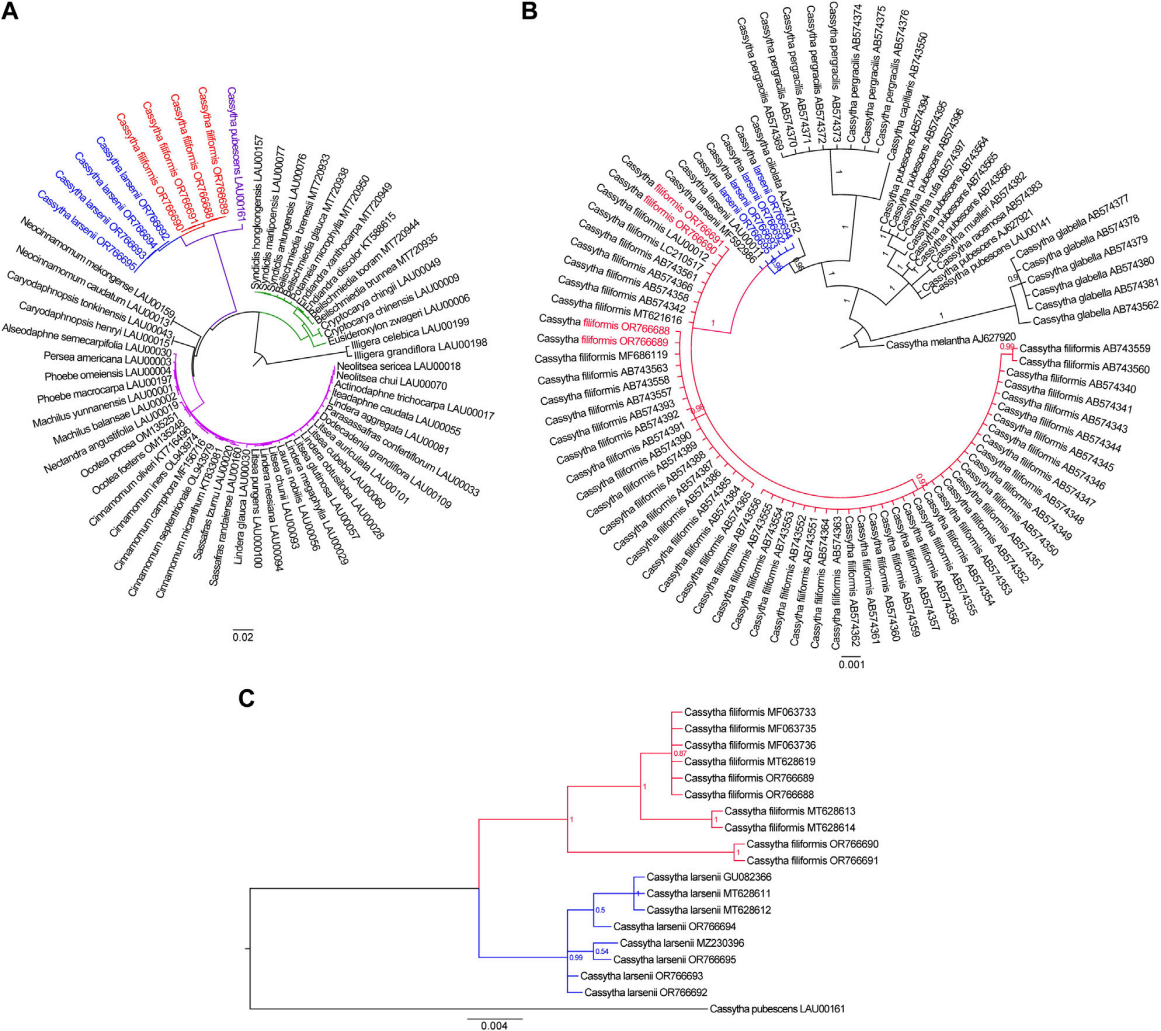
**(A)** The Maximum likelihood tree of 61 taxa of Laurales based on complete plastome sequences. Numbers at each node are bootstrap support values. **(B)** The Bayesian inference tree of 91 taxa of *Cassytha* species based on *trnK* sequences. The tree is rooted with *trnK* sequences of *C. melantha*. **(C)** The Bayesian inference tree of 19 taxa of *Cassytha* species based on ITS sequences. The tree is rooted with ITS sequences of *C. pubescens*.

To ascertain the phylogenetic placement of *Cassytha* species in relation to other members of the Lauraceae family with fully sequenced plastid genome sequences, we employed the complete plastomes of three *Cassytha* species to reconstruct phylogenetic relationships. We used two plastomes of *Illigera* species as out-groups. The phylogeny derived from the analysis of 61 complete plastid genome sequences is highly supported. Our phylogenetic analysis shows that the three *Cassytha* species form a sister clade to a group consisting of species belonging to the tribes Neocinnamomeae, Caryodaphnopsideae, and Laureae. The tribe Cryptocaryeae represents the next sister groups, followed by *Illigera* species (Figure 5A). The branch length in the maximum likelihood (ML) tree are 4.934 × 10^−4^ for four individuals of *C. filiformis* and 2.247 × 10^−3^ for four individuals of *C. larsenii*. To further investigate the phylogenetic relationships among the eight sequenced *Cassytha* individuals and other *Cassytha* taxa with reported barcoding data, we downloaded available *trnK* sequences from NCBI database. We included 91 *Cassytha* samples, with an outgroup accession of *C. melantha*, in the analysis of the data matrix with the length of 903 bp. The result of the Bayesian analysis shows that *C. filiformis* is sisters to *C. larsenii*, rather than *C. ciliolata* (Figure 5B). The branch lengths in the Bayesian inference (BI) tree are 1.997 × 10^−3^ for 57 individuals of *C. filiformis* and 5.902 × 10^−4^ for six individuals of *C. larsenii*. Finally, we downloaded available ITS sequences from GenBank and reconstructed the phylogeny consisted of 19 ITS sequences with a length of 579 bp. We used *C. pubescens* as an out-group (Figure 5C). The result of the Bayesian analysis shows that *C. filiformis* and *C. larsenii* individuals form two independent groups and the branch lengths are 1.204 × 10^−2^ for ten individuals of *C. filiformis* and 6.401 × 10^−3^ for eight individuals of *C. larsenii*.

### 3.6 Morphological characters

For *C. larsenii*, the scattered hairs on the haustoria were stable (Supplementary Figure S2A), and filiform hairs were observed on young stems (Supplementary Figure S2B) but not on annual and biennial stems (Supplementary Figure S2C), whereas stems with indumenta of simple, multicellular hairs were observed in two accessions of *C. filiformis* in China (Supplementary Figures S2G–I). For both *C. larsenii* and *C. filiformis*, the glabrous petal surfaces were observed with low magnification light microscopy (Supplementary Figures S2D, J), however, villous hairs were clearly observed on the edges of petals and petal surfaces in the electron microscopy photos (Supplementary Figure S2E, K). All members of genus *Cassytha* have fleshy fruits with a single seed, and the fruits of *C. filiformis* are oval with the mean size of 8 mm × 8 mm, while the fruits of *C. larsenii* are ellipsoid with the mean size of 5 mm × 7 mm. Compared with *C. larsenii*, *C. filiformis* has rounder and larger fruits (Supplementary Figure S2F, L).

## 4 Discussion

### 4.1 The extreme case of plastoma shrinkage in the family Lauraceae

This study produced eight complete plastid genomes for two species of the stem hemiparasitic genus *Cassytha*, which comprises nearly 23 species. The plastomes of *Cassytha* with the length of 114,215 ∼ 114,988 bp was significantly smaller than the other published plastomes in the family Lauraceae (Song et al., 2017b; Song et al., 2020). Three main reasons for these size differences were detected through comparative genomics analysis (Figure 3). First, one copy of the IR regions with the length of 24,717 bp was complete in *E. zwageri* but lost in the *Cassytha* plastomes with two segments, contributing almost 25 kb to the length difference. Second, the eight *Cassytha* plastomes have no six *ndh* genes including *ndhA*, *ndhC*, *ndhG*, *ndhI*, *ndhJ*, and *ndhK*. Two missing fragments with the length of 4 kb consist of three of the six genes, respectively, and their intergenic regions, which contributed around 8 kb to the length difference with *Cassytha* species. Third, five pseudogenes were detected in the eight *Cassytha* plastomes. Three *ndh* genes (*ndhB*, *ndhD*, and *ndhE*) were found to be pseudogenized, similar to *C. filiformis* (Wu et al., 2017). The length of ѱ*ndhB*, ѱ*ndhD*, ѱ*ndhE*, ѱ*ndhF*, and ѱ*ndhH* in *C. filiformis* are 1,191 bp, 428 bp, 159 bp, 302 bp, and 547 bp, respectively. In the plastome of *E. zwageri,* the length of the five *ndh* genes are 2,181 bp for *ndhB*, 1,508 bp for *ndhD*, 306 bp for *ndhE*, 2,229 bp for *ndhF*, and 1,182 bp for *ndhH*, which contributed around 7 kb to the length difference with *Cassytha* species.

### 4.2 The high sequence divergence among Lauraceae

Comparative genomic analysis indicated that there are 1,013 mutation sites including 24 micro-inversions, 249 indels, and 740 substitutions in the eight plastomes, which indicated that the nucleotide mutation sites in the plastomes of *Cassytha* species are more than that between species of *Machillus* (one micro-inversion, 65 indels, and 231 substitutions) and *Phoebe* (three micro-inversions, 73 indels, and 146 substitutions) (Song et al., 2015; Song et al., 2017a). The nucleotide variability values of the whole plastomes among the eight individuals from two *Cassytha* species were 0.34%, which approximates the nucleotide variability of five taxa (0.32%) (Song et al., 2016), 15 taxa (0.31%) (Liu et al., 2022), 18 taxa (0.37%) (Liu C. et al., 2021) in the tribe Laureae, and was much higher than the sequence divergence among three *Alseodaphne* species (0.12%) (Song et al., 2018), seven trinerved *Lindera* species (0.15%) (Tian et al., 2019), and seven *Ocotea* species (0.10%) (Trofimov et al., 2022).

### 4.3 Phylogeny of the sequenced *Cassytha* species and plastomes of Lauraceae

With species from 27 genera of Laurales, our phylogenomic analysis based on 61 plastid genomes supported a monophyletic *Cassytha* clade comprising species of *C. filiformis*, *C. larsenii*, and *C. pubescens*. Species of *Beilschmiedia*, *Cryptocarya*, *Endiandra*, *Eusideroxylon*, *Potameia*, *Sinopora*, and *Syndiclis*, formed the *Beilschmiedia*-*Cryptocarya* clade in the phylogeny, and the third clade including Neocinnamomeae, Caryodaphnopsideae, and Laureae species is separate from both the *Beilschmiedia*-*Cryptocarya* clade and *Cassytha* clade, as in previously published phylogenetic trees in the family Lauraceae (Song et al., 2017b; Song et al., 2020). The deep relationships of *Cassytha* taxa are separated into the following groups in our study. *C. melantha*, endemic to Australia, forms the first group in the phylogeny. *C. glabella*, endemic to Western Australia, forms the second group. The third group includes four Australia species *C. muelleri*, *C. pubescens*, *C. racemose*, and *C. rufa*. The fourth group includes two Asia species *C. capillaris* and *C. pergracilis*. And the last group includes an Africa species *C. ciliolata*, an Asia species *C. larsenii*, and the pantropical species *C. filiformis*. The phylogenetic placements of most groups are consistent with previously published phylogenetic relationships (Kokubugata et al., 2012), and the position of *C. larsenii* was firstly settled here in the way predicted from morphology.

### 4.4 Morphological difference among *Cassytha* specie

Although *C. ciliolata, C. filiformis*, and *C. larsenii* form the same group in the phylogeny, the persistence of hairs on stems and petal surfaces was used to distinguish the *Cassytha* species (Kokubugata et al., 2012). In *C. ciliolata*, filiform hairs on stems and glabrous or rufous-hispidulous twig tips were descripted on the basis of type specimens collected from mountains near Cape Town in Africa (Stapf, 1912). In *C. larsenii*, absence or sparsity of hairs on stems and glabrous or rufous-hispidulous twig tips were observed in all samples in China (Kostermans, 1994). In *C. filiformis*, it is remarkable that glabrous stems were observed not only in samples from Ryukyus of Japan and Taiwan of China but also in samples from Luzon Island and Rota Island in the Pacific (Kokubugata et al., 2012). However, stems with indumenta of simple, multicellular hairs were observed in *C. filiformis* samples from Australia, China, Japan, and Malaysia (Kokubugata et al., 2012). Therefore, glabrous stem is not a taxonomic key character for identifying *C. ciliolata*, *C. filiformis* and *C. larsenii*. Based on the type specimens, the glabrous petal surface was descripted as a common taxonomic character for *C. ciliolata*, *C. filiformis* and *C. larsenii*. However, villous petal surfaces and pubescent edge of petals were observed in the electron microscopy photos of *C. filiformis* and *C. larsenii*. Thus, presence or absence of hairs on petal surfaces is not appropriate as a taxonomic key character for identifying the three species. Finally, we suggest that, presence or absence of hairs on haustoria should be treated as key taxonomic evidence to distinguish *C. filiformis* and *C. larsenii.* Also, fruit shape or size can be a well distinction between the two species. Diversity of fruits may be related to growing in various geographical environments (Table 5).

**TABLE 5:** List of morphological traits of *Cassytha* species.

	*Cassytha larsenii* Kosterm.	*Cassytha filiformis* L.	*Cassytha ciliolata* Nees	*Cassytha pubescens* R.Br.
Habitat	mountain range	coastal	dry forests	dry forests
Stem color	straw-coloured	green to orange	yellow	dark green
Stem	glabrous	hairy or glabrous	filiform	glabrescent to pubescent
Petal surfaces	minutely rusty villous	glabrous	glabrous	pubescent
Fruit shape	ellipsoid	ovoid	globose or ellipsoid	globose to obovoid
Fruit size	4–6 mm × 6–8 mm	7–9 mm × 7–9 mm	4 mm × 4.5 mm	6–10 mm × 5.5–9 mm

### 4.5 New records for China

*Cassytha larsenii* Kosterm. was only known from Khun Yuam District, Thailand, this is the first record from China (Puer, Yunnan Province; Guangzhou, Guangdong Province; Linshui County, Hainan Province and Dongxing, Guangxi Province). It was found hosting as a hemiparasitic on the tree trunks of Fabaceae, Myrtaceae and Asteraceae at altitudes from 900 to 1900 m in the forest (Zhang et al., 2022). *C. larsenii* was observed flowering in June in Mojiang of Yunnan. Morphologically, it bears resemblance to *C. filiformis* due to hairs on petal surfaces, but differs from it in terms of the presence or absence of hairs on haustoria, shorter inflorescence, smaller flowers (0.5–0.75 mm) (Kostermans, 1994), and smaller fruits. Additionally, *C. filiformis* grows along coastal regions while *C. larsenii* is found in mountainous areas. Furthermore, strong evidence from our phylogenetic studies supports treating the new records of *C. larsenii* from China as a distinct species from *C. filiformis*. Using different analyses, in the present study we showed that the biodiversity of the genus *Cassytha* in China is underestimated, with more species than previously recognized (Li et al., 2008). And, evidences from plastid genome size, phylogenetics, and morphology characters suggest that at least two species partitions would require validation and formal description (Liu Z. F. et al., 2021). Our study provides important insights into the taxonomic, biodiversity, conservation biology, and phylogeographic of the genus *Cassytha*.

## 5 Conclusion

*Cassytha* is the only hemiparasitic vines in the Lauraceae family. Our study reports complete plastid genomes of two *Cassytha* species. 1,013 mutation sites, four large intragenomic deletions and five hotspots were found during comparative genomic research. Meanwhile, based on whole plastid, *trnK*, and ITS phylogenetic analyses respectively, confirmed a non-basal group comprising *C. filiformis*, *C. larsenii*, and *C. pubescens.* The position of *C. larsenii* was settled for the first time in accordance with presence or absence of hairs on the haustoria and the shape or size of fruits.

## Data Availability

The datasets presented in this study can be found in online repositories. The names of the repository/repositories and accession numbers can be found below: https://www.ncbi.nlm.nih.gov/, OR766688 to OR766695.
