# Supplementary material for: Comparative analyses of eight complete plastid genomes of two hemiparasitic Cassytha vines in the family Lauraceae
Source: Front Genet. 2023 Dec 13;14:1192170. doi: 10.3389/fgene.2023.1192170 (PMC10753772; doi:10.3389/fgene.2023.1192170)
Supplement: Supplementary file 1 [file Table1.docx]

Supplementary Material

Comparative analyses of eight complete plastid genomes of two hemiparasitic *Cassytha* vines in the family Lauraceae

Qun-Fei Yu, Yun-Hong Tan, Wen-Bin Yu, Shi-Ting Yang, Jie-Peng Huang, Marcos A. Caraballo-Ortiz, Chao Liu^*^, Yu Song^*^

# Supplementary Data

# FIGURE LEGENDS

**
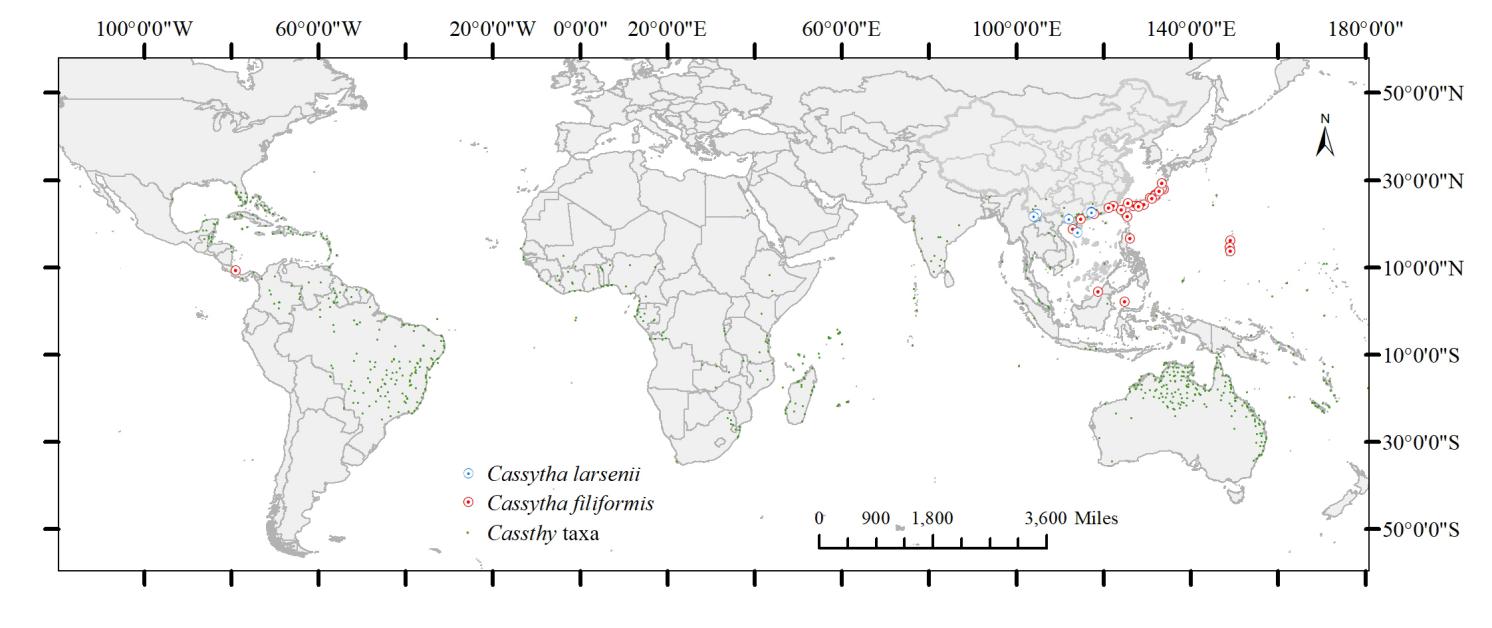
**

**FIGURE S1** The global distribution of *C. filiformis* and *C. larsenii*. Blue pots indicates *C. larsenii*, red pots indicates *C. filiformis* and small green pots indicates *Cassytha* taxa.


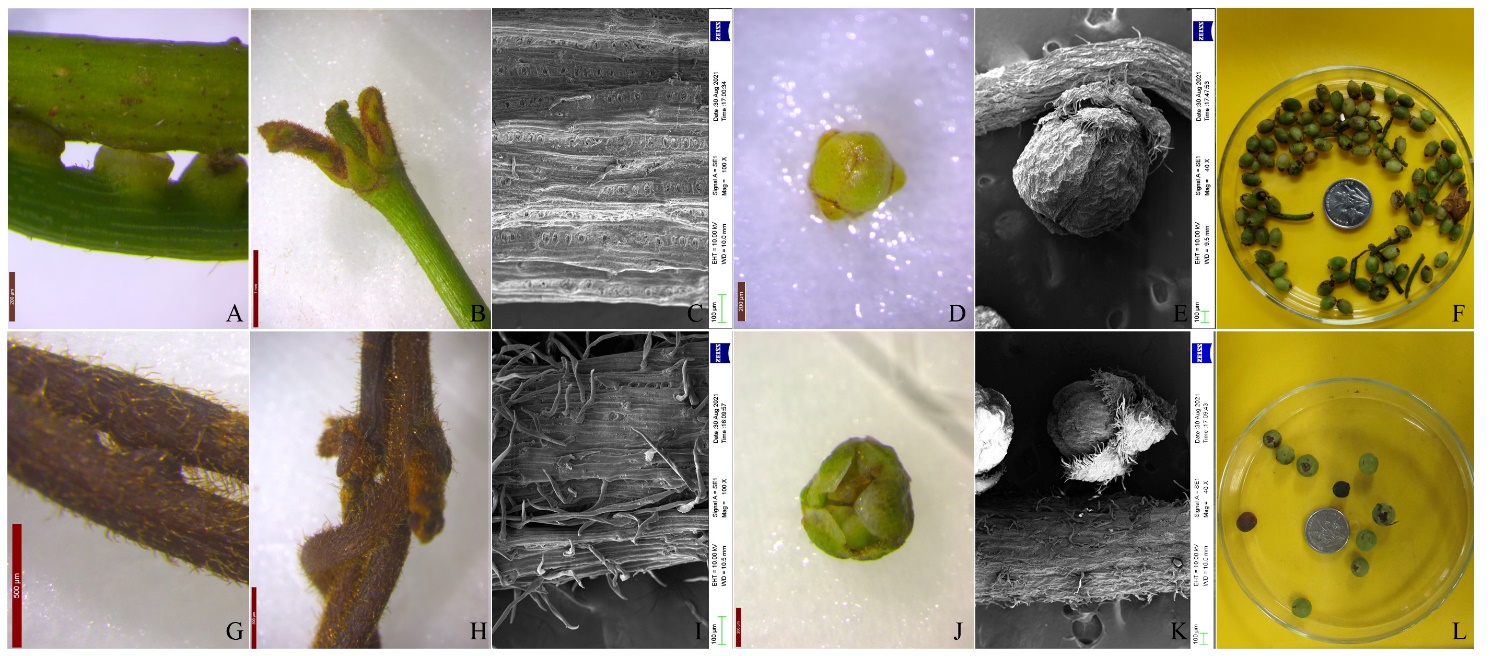


**FIGURE S2** Haustoria, Stems, Petal surfaces and Fruits of *C. larsenii* and *C. filiformis* under SM and SEM, respectively. A-C. Haustoria, Stems of *C. larsenii.* D, E. Petal surfaces of *C. larsenii.* F. Fruits of *C. larsenii.* G-I. Haustoria, Stems of *C. filiformis.* J, K. Petal surfaces of *C. filiformis.* L. Fruits of *C. filiformis.*

**TABLE S1** Forms and numbers of indel mutational events in the plastid genome sequences between the species of *C. filiformis* and *C. larsenii*.

| Position site | Driection* | Motif | Type |
| --- | --- | --- | --- |
| 92 | Deletion | aatggtatataaaatgccaatcaatccctttacttaaaaaaaagggttttgaataacatagtatacaaacgctttattagattgaataaaag | nonSSR |
| 1852 | Deletion | t | SSR |
| 2827 | Deletion | taaaaaaag | nonSSR |
| 4331 | Deletion | ttttttgtttttttt | SSR |
| 4503 | Insertion | aatat | nonSSR |
| 4526 | Deletion | a | SSR |
| 4791 | Insertion | t | SSR |
| 4849 | Deletion | aaaaaaaaaaaagaa | SSR |
| 5293 | Deletion | t | SSR |
| 5346 | Deletion | a | SSR |
| 5468 | Deletion | t | SSR |
| 5731 | Insertion | t | SSR |
| 6236 | Deletion | a | SSR |
| 6319 | Deletion | a | SSR |
| 6712 | Deletion | t | SSR |
| 6776 | Deletion | t | SSR |
| 6921 | Deletion | a | SSR |
| 7001 | Insertion | g | nonSSR |
| 7108 | Insertion | t | SSR |
| 7145 | Insertion | g | SSR |
| 7207 | Deletion | ttgttatgattctacaca | nonSSR |
| 7406 | Insertion | a | nonSSR |
| 7687 | Insertion | a | SSR |
| 8229 | Insertion | ttcgaaa | nonSSR |
| 8236 | Insertion | t | SSR |
| 8259 | Insertion | catttga | nonSSR |
| 8455 | Insertion | t | SSR |
| 8597 | Deletion | t | SSR |
| 8687 | Deletion | a | SSR |
| 8694 | Insertion | aggc | nonSSR |
| 8743 | Insertion | t | SSR |
| 9499 | Insertion | t | SSR |
| 9609 | Deletion | a | SSR |
| 9912 | Insertion | t | SSR |
| 9915 | Insertion | g | nonSSR |
| 10133 | Deletion | t | SSR |
| 10253 | Deletion | a | SSR |
| 10406 | Deletion | a | SSR |
| 12799 | Deletion | a | SSR |
| 12863 | Insertion | tatt | SSR |
| 13126 | Deletion | a | SSR |
| 13402 | Deletion | t | SSR |
| 13531 | Deletion | a | SSR |
| 13741 | Deletion | t | SSR |
| 14032 | Deletion | a | SSR |
| 14246 | Deletion | a | SSR |
| 14269 | Deletion | ggaatctatcgaaattccgtgggatgggaaaacatatagttaacatatataactag | nonSSR |
| 14483 | Deletion | t | SSR |
| 14502 | Deletion | a | SSR |
| 16231 | Deletion | t | SSR |
| 16258 | Insertion | a | SSR |
| 22474 | Deletion | aat | SSR |
| 22829 | Deletion | t | SSR |
| 27206 | Deletion | a | SSR |
| 27322 | Insertion | tatcgaagtccccttgaggcaacata | nonSSR |
| 27395 | Deletion | a | SSR |
| 27749 | Insertion | t | SSR |
| 27791 | Insertion | a | SSR |
| 27956 | Deletion | ataagtaatagaaatatagt | nonSSR |
| 28436 | Deletion | t | SSR |
| 28501 | Deletion | a | SSR |
| 29086 | Deletion | a | SSR |
| 29165 | Insertion | acttga | nonSSR |
| 29316 | Deletion | ta | SSR |
| 29623 | Deletion | a | SSR |
| 30062 | Deletion | t | SSR |
| 30075 | Deletion | a | nonSSR |
| 30554 | Deletion | t | SSR |
| 30924 | Deletion | a | SSR |
| 31033 | Insertion | cgaca | SSR |
| 31325 | Insertion | t | SSR |
| 31515 | Insertion | t | SSR |
| 32037 | Deletion | at | SSR |
| 32558 | Deletion | t | SSR |
| 32580 | Deletion | a | SSR |
| 33405 | Insertion | g | SSR |
| 33460 | Insertion | aaaaaaatagagt | nonSSR |
| 33473 | Deletion | ctttttt | SSR |
| 33493 | Insertion | ctacccgttaaaagatatactctgtaattttagatttattcgaaggaaatataacaaagaagacaataaaaaaagcaataaaaaaagaaagagtaacagattctctattatctatagactactgtattagtttagtatacacaaaaataaggagaatatataaagatattgattactcttttctaaatatcaccaataaataatatccaagaataagatgaatttatgtaccggtgagttagatacgggaagtaatgacgataaaaaggatcacttttttactgacatttatt | nonSSR |
| 33786 | Deletion | a | SSR |
| 36658 | Deletion | g | SSR |
| 37015 | Deletion | a | SSR |
| 37040 | Deletion | a | SSR |
| 37416 | Insertion | t | SSR |
| 37539 | Deletion | a | SSR |
| 37915 | Deletion | a | SSR |
| 38596 | Insertion | a | SSR |
| 43210 | Deletion | c | SSR |
| 43365 | Deletion | t | SSR |
| 43587 | Insertion | g | SSR |
| 43609 | Deletion | ttatt | SSR |
| 43644 | Deletion | a | SSR |
| 43954 | Deletion | t | SSR |
| 44303 | Deletion | a | SSR |
| 44319 | Deletion | t | nonSSR |
| 45325 | Insertion | g | nonSSR |
| 45891 | Deletion | aatcc | SSR |
| 46298 | Insertion | t | SSR |
| 46324 | Deletion | a | SSR |
| 46692 | Deletion | ag | SSR |
| 47735 | Deletion | a | SSR |
| 47751 | Deletion | g | SSR |
| 48653 | Deletion | t | SSR |
| 49763 | Deletion | t | SSR |
| 50371 | Insertion | t | SSR |
| 50572 | Deletion | t | SSR |
| 52776 | Deletion | t | SSR |
| 54817 | Deletion | tatac | SSR |
| 55127 | Deletion | tatatgatataactctt | nonSSR |
| 55159 | Insertion | aatagaagaaagagttatatcatatatagttttttcctcggttcccgcaaaaccatttatttctttc | nonSSR |
| 55167 | Insertion | aaagagttatatcatat | nonSSR |
| 56790 | Deletion | t | SSR |
| 56906 | Deletion | a | SSR |
| 57182 | Deletion | a | SSR |
| 57473 | Insertion | g | SSR |
| 57539 | Insertion | a | SSR |
| 57770 | Insertion | a | SSR |
| 58776 | Deletion | t | SSR |
| 59137 | Deletion | t | SSR |
| 59218 | Deletion | a | SSR |
| 61525 | Deletion | a | SSR |
| 61788 | Insertion | a | SSR |
| 61885 | Insertion | t | SSR |
| 62198 | Deletion | t | SSR |
| 63264 | Insertion | ttcctttc | SSR |
| 63942 | Deletion | a | SSR |
| 63974 | Insertion | t | SSR |
| 63976 | Insertion | ttatgccatggttactccaaaagaattggatttttgaatgaagttataagacacagttcttattattattattactttactcacaaattgctgactgaatccgttgatttgattgaaatcacgtgatcca | nonSSR |
| 64105 | Insertion | gatt | nonSSR |
| 64158 | Deletion | t | SSR |
| 64277 | Deletion | a | SSR |
| 64481 | Deletion | a | SSR |
| 64773 | Insertion | t | SSR |
| 65201 | Deletion | t | SSR |
| 65697 | Deletion | aagggatcaa | nonSSR |
| 65947 | Insertion | cttcg | nonSSR |
| 66263 | Deletion | at | SSR |
| 66775 | Deletion | a | SSR |
| 66836 | Deletion | t | SSR |
| 66855 | Deletion | att | SSR |
| 66900 | Deletion | ttatcatatgaatttctacta | nonSSR |
| 67518 | Deletion | t | SSR |
| 68872 | Insertion | a | SSR |
| 68972 | Deletion | a | SSR |
| 68987 | Deletion | t | SSR |
| 69089 | Insertion | a | SSR |
| 69130 | Deletion | gat | SSR |
| 69297 | Deletion | t | SSR |
| 69649 | Insertion | a | SSR |
| 69719 | Deletion | a | SSR |
| 69771 | Deletion | t | SSR |
| 70053 | Deletion | t | SSR |
| 70650 | Deletion | t | SSR |
| 72463 | Insertion | ttgc | SSR |
| 73856 | Insertion | a | SSR |
| 74921 | Deletion | tgtt | SSR |
| 75281 | Deletion | a | SSR |
| 75677 | Insertion | g | SSR |
| 75677 | Insertion | g | SSR |
| 76410 | Deletion | t | SSR |
| 76431 | Deletion | a | SSR |
| 79080 | Deletion | t | SSR |
| 79581 | Deletion | t | SSR |
| 79688 | Insertion | aaat | nonSSR |
| 79691 | Insertion | a | SSR |
| 80158 | Insertion | t | SSR |
| 80309 | Insertion | a | SSR |
| 80885 | Deletion | t | SSR |
| 80897 | Insertion | a | SSR |
| 81029 | Deletion | t | SSR |
| 81154 | Deletion | t | SSR |
| 81897 | Insertion | t | SSR |
| 82739 | Deletion | t | SSR |
| 82768 | Deletion | a | SSR |
| 83243 | Deletion | t | SSR |
| 84304 | Deletion | t | SSR |
| 84467 | Deletion | tatatc | SSR |
| 84708 | Insertion | aatagtgcaatgaagatgagttcgacatttgacaatagaagtctattgactttgacccaataaccgagtacaatggtgtaatgaagatgagttcgacaaaatgctttatttctgtcctagttgatcctaatttgacataaggctattgactttgacccaataaccgattac | nonSSR |
| 92242 | Insertion | g | SSR |
| 92915 | Insertion | actgc | nonSSR |
| 93163 | Deletion | a | SSR |
| 93677 | Insertion | t | SSR |
| 93709 | Deletion | a | SSR |
| 93825 | Deletion | t | SSR |
| 93934 | Deletion | c | SSR |
| 93942 | Deletion | t | SSR |
| 93957 | Deletion | a | SSR |
| 94038 | Deletion | t | SSR |
| 94246 | Deletion | a | SSR |
| 94277 | Deletion | t | SSR |
| 94342 | Deletion | a | SSR |
| 94492 | Deletion | t | SSR |
| 94711 | Insertion | atatctta | SSR |
| 94786 | Insertion | ta | SSR |
| 95916 | Deletion | t | SSR |
| 95983 | Insertion | c | SSR |
| 95985 | Deletion | t | SSR |
| 96268 | Deletion | ta | nonSSR |
| 96275 | Insertion | c | nonSSR |
| 96495 | Insertion | a | SSR |
| 96788 | Insertion | tactgt | nonSSR |
| 96969 | Deletion | a | SSR |
| 97088 | Deletion | a | SSR |
| 97118 | Deletion | atccta | SSR |
| 97404 | Deletion | a | SSR |
| 97759 | Deletion | t | SSR |
| 97857 | Insertion | t | SSR |
| 98150 | Insertion | t | SSR |
| 98294 | Insertion | caaag | nonSSR |
| 99045 | Deletion | c | SSR |
| 99570 | Deletion | t | SSR |
| 103690 | Deletion | catttgtttgatcatttgtatcatttgtttgatcatttggat | nonSSR |
| 103732 | Insertion | cagttgtttgatcatttggat | SSR |
| 104658 | Insertion | tcttccatttcttccatt | nonSSR |
| 105437 | Insertion | ccctttttttgtttattgcaattttattattattaatttaatattattatataataatttcttcactttccatattaagtataatagattaaaaaaaaaaaaaaaaagacttcaaaagcatcacaaacct | nonSSR |
| 105447 | Insertion | g | nonSSR |
| 105527 | Insertion | a | SSR |
| 105531 | Insertion | a | nonSSR |
| 105544 | Insertion | g | nonSSR |
| 105594 | Insertion | t | SSR |
| 105623 | Insertion | t | nonSSR |
| 105681 | Deletion | gtaaa | SSR |
| 105877 | Deletion | c | SSR |
| 106090 | Deletion | gtctttgtttctattgcattctatctcatcacatcacattatgttgtac | nonSSR |
| 106286 | Insertion | ccacaattaccacgagcaaacata | nonSSR |
| 106389 | Insertion | ggagttatggggagtaaagcaaaaatat | nonSSR |
| 106529 | Insertion | tcttgatcatggttttcctacccccagaggtaaaggaaaggtcct | nonSSR |
| 106746 | Insertion | ttcg | SSR |
| 106859 | Insertion | t | SSR |
| 107152 | Insertion | t | SSR |
| 110400 | Insertion | atacc | nonSSR |
| 110715 | Deletion | t | SSR |
| 111138 | Deletion | c | SSR |
| 111885 | Deletion | gaggatt | nonSSR |
| 111940 | Deletion | a | SSR |
| 112350 | Insertion | g | nonSSR |
| 114781 | Insertion | ataagatgatacggcccctattcttgcgccaaaaatcttctcatttacgaaagaactggagatacatctcttttcaattttcattcaagagttcttatgtgtttccacgcccttttgatatgtcaaaaatagaaaaattacttttgttaggaacatatacaagatttgttactac | nonSSR |
| 115674 | Deletion | a | SSR |
| 116578 | Insertion | atactctatctcatagagacat | nonSSR |
| 116742 | Insertion | g | nonSSR |
| 117372 | Deletion | a | SSR |
| 117677 | Deletion | t | SSR |
| 117973 | Deletion | t | SSR |
| 118013 | Insertion | t | SSR |
| 118297 | Insertion | a | SSR |
| 118477 | Deletion | t | nonSSR |
| 118684 | Deletion | tc | nonSSR |
| 118686 | Deletion | t | SSR |
| 118770 | Insertion | cttttatcactta | nonSSR |

*The plastome of *C. filiformis* (OR766688) was used as a reference.
